# Influencing factors and mechanisms promoting proactive health behavior intention: an integration of the health belief model and the theory of planned behavior

**DOI:** 10.3389/fpubh.2025.1629046

**Published:** 2025-07-10

**Authors:** Lidong Fang, Qiaoqiao Zhang, Ning Zhou, Jin Chen, Hu Lou

**Affiliations:** School of Sports Science, Nantong University, Nantong, China

**Keywords:** proactive health, behavioral intention, theory of planned behavior (TPB), health belief model, self-efficacy

## Abstract

**Background:**

Promoting proactive health behaviors is an effective strategy for addressing public health challenges and advancing the “Healthy China” initiative. This study aims to explore the driving factors and mechanisms influencing proactive health behavior intention by integrating the theory of planned behavior (TPB) and the health belief model (HBM).

**Methods:**

A cross-sectional survey design was employed. A structured questionnaire was developed based on the theory of planned behavior (TPB) and the health belief model (HBM), covering eight dimensions: health behavior attitude, subjective norms, perceived behavioral control, self-efficacy, perceived susceptibility, perceived severity, perceived benefits, and perceived barriers. A total of 462 valid responses were collected using convenience sampling at a hospital health examination center in Jiangsu Province, China. Participants were approached on-site during routine check-ups and voluntarily completed the survey after providing informed consent. Data were analyzed using SPSS 26.0 and AMOS 26.0. Reliability and validity were tested using Confirmatory Factor Analysis (CFA), and hypotheses were examined through Structural Equation Modeling (SEM).

**Results:**

The findings revealed that perceived susceptibility, severity, benefits, and barriers significantly influenced individuals’ attitudes toward health behaviors. Attitudes, subjective norms, and perceived behavioral control significantly predicted intention, with self-efficacy partially mediating these effects. Perceived barriers had a negative effect, suggesting practical challenges hinder the development of health intentions.

**Conclusion:**

Health belief factors, especially perceived benefits, significantly influence health behavior attitude. TPB variables—particularly attitude—are key predictors of proactive health behavior intention. Self-efficacy acts as an important mediator, enhancing the explanatory power of the integrated TPB-HBM model. These findings provide theoretical and practical guidance for designing interventions to promote proactive health behavior in the general population.

## 1 Introduction

The rising global burden of chronic and lifestyle-related diseases poses a serious threat to national health systems. Although countries like the United States and China have seen dramatic increases in healthcare spending over the past decades, these investments have not effectively curbed the prevalence of preventable diseases (1, 2). In China alone, lifestyle-related illnesses now account for nearly 89% of all deaths (3), underscoring a growing mismatch between medical spending and population health outcomes. This reality highlights the urgent need to shift from a reactive, treatment-centered model to a proactive health behavior approach that emphasizes prevention and individual responsibility. Previous studies have shown that proactive health behaviors—such as regular exercise, healthy diet, and stress regulation—can significantly reduce chronic disease risks and improve quality of life outcome (4). Against this backdrop, promoting proactive health behaviors—defined as voluntary, health-enhancing actions taken before disease onset—has become a crucial strategy for improving population health and ensuring the sustainability of health systems.

In alignment with this national policy shift toward prevention, theoretical models that explain health-related decision-making are essential for guiding behavioral interventions. Two of the most widely used frameworks in this context are the health belief model (HBM) and the theory of planned behavior (TPB). HBM posits that individuals’ health actions are primarily influenced by their perceived susceptibility to a disease, perceived severity of its consequences, perceived benefits of taking action, and perceived barriers to doing so. In contrast, TPB emphasizes the role of behavioral intention, which is shaped by one’s attitude toward the behavior, perceived social norms, and perceived behavioral control.

While both models offer valuable insights independently, their integration allows for a more comprehensive understanding of health behavior formation. Specifically, combining HBM’s emphasis on risk perception and motivation with TPB’s focus on intention and volitional control enhances explanatory power and practical relevance. In light of this, the present study adopts an integrated HBM-TPB framework to investigate the key factors influencing proactive health behavior intention. This approach not only aligns with the preventive emphasis of the Healthy China Initiative but also contributes to the theoretical advancement and practical design of public health interventions aimed at encouraging individual health responsibility (5).

Therefore, this study aims to investigate the key factors and mechanisms influencing proactive health behavior intention by integrating the health belief model and the theory of planned behavior, providing theoretical insights and practical guidance for promoting health-oriented behavior change.

## 2 Theoretical analysis and research hypotheses

### 2.1 Theoretical analysis

Conceptually, proactive health behavior can be understood through the lens of complexity science, which views the human body as a dynamic system capable of adaptation. By actively introducing manageable lifestyle stimuli—such as exercise, diet, or stress management—the body’s regulatory systems can self-adjust and optimize toward improved health outcomes (6). The connotation of proactive health behavior includes four dimensions: practice concept, participating subjects, implementation path, and health results (7). This practical activity and medical model draws from the concepts of holistic medicine and the treatment of unhealthy diseases in Chinese medicine, leveraging modern science and technology, and aligning with government-led initiatives (8). It fosters a societal and individual mindset oriented toward proactive health behavior, aiming to reduce the incidence of chronic diseases and improve psychological well-being, particularly among working-age adults by implementing health interventions, fostering healthy habits, and creating a conducive environment. Proactive health behavior is defined as an individual’s proactive engagement in health management, aimed at achieving sustained health capacity, a healthy and optimal quality of life, and effective social adaptability. This concept encompasses the individual’s initiative to acquire health information and select health behaviors. The promotion of public physical and mental health by sports constitutes a paradigmatic instance of proactive health behavior. Consequently, proactive health behavior can be defined as the external activity of individuals in the process of coping with diseases and health management, with the objective of meeting health needs, consciously assuming personal health responsibility, fully using available health resources, and exerting subjective initiative to manage risk factors such as labor, rest, diet, exercise, and emotion.

The theory of planned behavior (TPB) is a theoretical framework that predicts and explains individual behavior, with a focus on behaviors adopted after deliberation. The classical theory of planned behavior posits a five-factor model comprising behavior, behavioral intention, behavioral attitude, subjective norm, and perceived behavioral control. According to Ajzen (9), all factors that may influence behavior are indirectly influenced by behavioral intention, which is the proximal determinant of behavior. In the presence of adequate conditions of actual control, behavioral intention directly determines behavior. Meanwhile, behavioral intention is influenced by three related factors: the more positive the attitude, the greater the support from significant others, and the stronger the perceived behavioral control, then the greater the behavioral intention, and vice versa (10). However, the theory of planned behavior model is not without its limitations. In certain research contexts, additional factors may need to be considered to enhance the theory of planned behavior. This enhancement can be achieved by extending the model to different types of behaviors, samples, and research objectives. Doing so can improve the model’s ability to predict and explain specific behaviors (11).

The health belief model (HBM) is an explanatory theory proposed by the research fields of preventive medicine and nursing. The HBM is applied to disease screening and prevention, health behavior management, and intervention of adverse health behaviors. The HBM assumes that individuals have specific belief systems that induce health-related behaviors. The model includes perceived susceptibility, perceived seriousness, perceived benefits, and perceived barriers (12). Perceived susceptibility, defined as the perceived likelihood of experiencing a health problem, is a fundamental component of the health belief model. Perceived severity, encompassing the perceived magnitude of a health problem and its potential consequences, is another crucial element. Perceived benefits, referring to the belief that adopting a health behavior is effective in preventing or mitigating a disease, is also a pivotal component. Finally, perceived barriers refer to the obstacles or difficulties that an individual believes they will encounter in performing a health behavior. The health belief model has been employed in studies examining various health behaviors, including physical activity (13), vaccination (14), and cancer screening (15).

**Figure 1:**
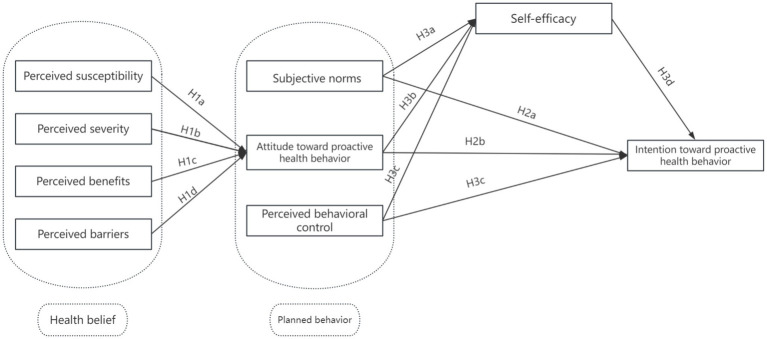
The proactive health behavior theoretical model based on the health belief model and theory of planned behavior.

However, the theory of planned behavior, while widely applied, primarily addresses volitional and intention-driven behavior but tends to overlook individuals’ perceptions of health threats and the motivational triggers derived from risk assessment. In contrast, the health belief model captures these early motivational beliefs—such as perceived susceptibility and severity—that can initiate the cognitive process leading to behavioral intention. Therefore, integrating HBM with TPB allows the model to account for both motivational precursors (HBM) and volitional processes (TPB), offering a more comprehensive understanding of proactive health behavior formation. This integration is especially relevant in preventive contexts, where individuals often decide whether to act in the absence of symptoms or immediate threats (16, 17). While several studies have applied either the health belief model or the theory of planned behavior to predict various health behaviors, few have explicitly examined their integrated use in the context of proactive health behavior—particularly within the Chinese population (18, 19). Therefore, this study incorporates health beliefs as an extension variable of the theory of planned behavior, proposes related hypotheses, and constructs the SEM model of proactive health behaviors (Figure 1), thus providing theoretical support for further clarifying the formation process of active health behavior.

In this study, the term “mechanisms” refers to the psychological and cognitive pathways through which health beliefs (e.g., perceived susceptibility, perceived benefits, perceived barriers) and behavioral constructs (e.g., attitude, subjective norms, perceived behavioral control) influence individuals’ intention to engage in proactive health behavior. These mechanisms are theoretically grounded in the integration of the health belief model and the theory of planned behavior, and empirically operationalized in the structural equation model by identifying both direct and indirect paths—notably, the mediating role of self-efficacy. This approach allows us to elucidate how these factors work together to promote the formation of proactive health behavior intention.

### 2.2 Research hypotheses

Based on the above theoretical analysis, the health belief model is considered a precursor to the theory of planned behavior. This integrated model incorporates key variables such as perceived susceptibility, perceived severity, perceived benefits, and perceived barriers (from HBM), as well as health behavior attitude, subjective norms, perceived behavioral control, self-efficacy, and behavioral intention (from TPB). These variables reflect both individual cognitive assessments and motivational determinants of health-related actions. To test the hypothesized relationships among these constructs, this study employs Structural Equation Modeling (SEM), a robust statistical technique suitable for examining complex, multi-variable theoretical frameworks. SEM is particularly appropriate for this research because it allows for simultaneous analysis of direct and indirect effects among latent variables, and effectively captures the mediating role of self-efficacy. Based on this theoretical foundation, the following research hypotheses are proposed:

*H1:* Health belief variables positively impact attitudes toward proactive health behavior.

*H1a:* Perceived susceptibility positively impacts attitudes toward proactive health behavior.

*H1b:* Perceived severity positively impacts attitudes toward proactive health behavior.

*H1c:* Perceived benefits positively impact attitudes toward proactive health behavior.

*H1d:* Perceived barriers have a negative impact on attitudes toward proactive health behavior.

The theory of planned behavior is one of the most widely applied behavioral explanation theories. Behavioral attitudes, subjective norms, and perceived behavioral control have been proven to influence behavior. For example, Liang Jinhui’s study found that attitudes toward physical activity, subjective norms, and perceived behavioral control all positively and significantly affect adolescents’ intentions to engage in ice and snow sports. A study by Wang et al. (20) also showed that attitudes toward physical activity, subjective norms, and perceived behavioral control positively and significantly influence university students’ intentions to engage in physical activity and, through intention, further impact physical activity behavior. A meta-analysis by Hagger et al. (21) on perceived behavioral control and health behavior found that perceived behavioral control can significantly and positively influence health behavior intentions.

Based on the above research findings, the following hypotheses are proposed:

*H2*: The independent variables of the theory of planned behavior positively impact proactive health behavior intention.

*H2a*: Attitude has a significant positive impact on proactive health behavior intention.

*H2b*: Subjective norm has a significant positive impact on proactive health behavior intention.

*H2c*: Perceived behavioral control has a significant positive impact on proactive health behavior intention.

Self-efficacy refers to an individual’s perception of the difficulty or confidence in performing a particular behavior. Both the health belief model and the theory of planned behavior recognize the critical role of self-efficacy, assuming that an individual’s subjective initiative is a crucial factor in triggering specific behaviors. Existing studies have shown that self-efficacy can positively influence an individual’s behavioral intention, and health management interventions based on the theory of planned behavior can enhance an individual’s self-efficacy. Therefore, the study further hypotheses:

*H3*: Self-efficacy mediates the relationship between the theory of planned behavior variables and proactive health behavior intention.

## 3 Research methodology

### 3.1 Questionnaire design and measurement method

The integration of TPB and HBM in this study is theoretically justified by their complementary strengths. While TPB excels at explaining volitional, intention-driven behaviors, it lacks constructs related to early-stage risk perception and threat appraisal. In contrast, HBM focuses on motivational precursors—such as perceived susceptibility and perceived severity—but does not elaborate on how intention translates into action. By combining these models, this study captures both the motivational phase (HBM) and the volitional phase (TPB) of proactive health behavior formation. This integrated approach offers a more comprehensive and dynamic framework than either model alone, particularly in the context of preventive health actions, where individuals often act without immediate symptoms. Moreover, this study advances prior work by operationalizing the integrated model within the emerging construct of “proactive health behavior,” which remains underexplored in current literature (22, 23).

Based on the above research hypotheses, this study combines the health belief model and the theory of planned behavior. A proactive health behavior survey questionnaire was designed to collect sample data, referencing existing health beliefs and theory of planned behavior questionnaires. The questionnaire consists of three main parts: The first part is the introduction, which aims to explain the purpose of the survey and provide guidance for the respondents in completing the questionnaire. The second part involves basic information about the respondents, such as gender, age, and average monthly income. The third part is the main body of the questionnaire, which includes the measurement items of the “Proactive Health Behavior” model (Table 1). A 7-point Likert scale is used for measurement. The measurement of proactive health behavior intention was guided by the theory of planned behavior and followed Ajzen’s item construction principles. This construct was operationalized as the individual’s expressed willingness and concrete plans to engage in preventive and health-enhancing behaviors over the next 3 months. The three items used were adapted from validated TPB-based health behavior questionnaires in existing literature, and were reviewed by two public health experts to ensure content validity. Pilot testing further confirmed the items’ clarity, comprehensibility, and alignment with the intended construct. The positive and reverse scoring items were randomly set during the questionnaire design to avoid random responses from participants. These reverse-coded items were pre-tested in the pilot phase to ensure clarity and avoid cognitive confusion. Feedback from participants was used to revise ambiguous wording. During data processing, all reverse-coded items were correctly reverse-scored prior to statistical analysis to ensure reliability and consistency in the results.

**Table 1:** Measurement items of the driving factors of proactive health behavior intention.

Theory	Variable	Measurement items
Theory of planned behavior	Behavioral attitude	1. I believe that improving health behaviors can bring long-term benefits.
		2. Pursuing health goals is an essential part of my personal development.
		3. Maintaining a healthy lifestyle is worth the time and effort.
		4. I have a positive attitude toward participating in health promotion activities.
	Subjective norms	1. Most essential people support my proactive efforts over the next three months to continuously challenge myself in physical training, competitions, or other physical activities, actively manage my diet, sleep, and emotions, and choose a healthy lifestyle. (My family/friends/colleagues encourage me to engage in proactive health behaviors.).
		2. Most people, like me, will take proactive actions over the next three months to continuously challenge themselves in physical training, competitions, or other physical activities, actively manage their diet, sleep, and emotions, and choose a healthy lifestyle. (I believe others significantly influence my health behaviors.).
		3. I feel that health behaviors are valued in my social circle.
	Perceived behavioral control	1. Over the next three months, I am confident that I can take proactive actions to continuously challenge myself in physical training, competitions, or other physical activities, actively manage my diet, sleep, and emotions, and choose a healthy lifestyle. (I believe I can maintain healthy choices when facing temptations.).
		2. Over the next three months, I will take proactive actions to continuously challenge myself in physical training, competitions, or other physical activities, actively manage my diet, sleep, and emotions, and choose a healthy lifestyle, depending on myself. (I believe I can engage in proactive health behaviors.).
		3. I feel confident in coping with health management challenges.
	Behavioral intention	1. Over the next three months, I plan to take proactive actions to continuously challenge myself in physical training, competitions, or other physical activities, actively manage my diet, sleep, and emotions, and choose a healthy lifestyle. (I plan to engage in proactive health behaviors in the coming months, such as actively managing my diet, sleep, and emotions.).
		2. I plan to undergo regular health check-ups to monitor my health status.
		3. I intend to set health goals and work towards achieving them, and I plan to share information about healthy lifestyles with my family and friends.
Mediating variable	Self-efficacy	1. I know how to engage in proactive health behaviors. (For example, I am confident that I can exercise at least three times a week / I believe I can resist the temptation of unhealthy foods.).
		2. Engaging in proactive health behaviors is easy if I want to.
		3. I can overcome the barriers to implementing health behaviors.
Health belief model	Perceived susceptibility	1. I have or worry about experiencing some physical or mental health issues.
		2. I feel that I am in a state of subhealth.
		3. I believe that unhealthy lifestyle habits directly impact my health.
		4. I realize I need to pay more attention to my health to avoid potential diseases.
	Perceived severity	1. My health issues have caused inconveniences in my work life.
		2. My health issues have caused trouble for my family and those around me.
		3. I believe that health issues may affect my mental health and emotional stability.
		4. I believe that chronic diseases can have a long-term impact on my physical health.
	Perceived benefits	1. Proactive health behaviors make me feel energized.
		2. Proactive health behaviors can improve my health status (for example, enhancing immunity and reducing the risk of illness).
		3. I believe that maintaining a healthy weight has significant benefits for my physical and mental health.
	Perceived barriers	1. I am too lazy to engage in proactive health behaviors.
		2. I find it challenging to stick to proactive health behaviors.
		3. I do not have enough time to engage in health activities.
		4. The surrounding environment does not support me in health activities.

To ensure content validity, the questionnaire items were reviewed by a panel of three experts in health psychology and behavioral science. These experts evaluated the relevance, clarity, and representativeness of each item in relation to the constructs being measured. Modifications were made based on their feedback, particularly for items that showed ambiguity or potential overlap. A pilot test with 30 participants was then conducted to assess item clarity and readability. Based on their responses and open-ended feedback, several wording adjustments were made to enhance comprehensibility. The final version of the questionnaire demonstrated satisfactory content coverage and internal consistency, as confirmed by CFA and reliability analysis.

For the relevant items of the theory of planned behavior questionnaire, the method recommended by Ajzen (24) was used for constructing the questionnaire. First, the operational definition of “proactive health behavior” is the individual’s subjective initiative to actively manage external activities related to risk factors such as work, rest, diet, exercise, and emotions. Then, four dimensions—behavioral attitude, subjective norm, perceived behavioral control, and proactive health behavior intention—were developed for specific measurement. The self-efficacy measurement refers to the three-item design used by Tarker (25) in the study that integrated the health belief model and the theory of planned behavior. The perceived barriers and perceived benefits items in the health belief model questionnaire were adapted from the items used by Wu et al. (26) in their research on the relationship between the health belief model and physical activity. The items for perceived susceptibility and perceived severity were adapted from the studies of Hosseini et al. (13) that explored the influencing factors of physical activity using the health belief model.

### 3.2 Data collection and survey participants

This study adopted a cross-sectional design. A convenience sampling method was used to recruit participants from a hospital health check-up center in Jiangsu Province during October 2024. Participants were eligible if they were aged 25–60, literate in Chinese, and willing to provide informed consent. Questionnaires were administered both online and in-person.

Before the formal distribution of the questionnaire, 30 questionnaires were firstly distributed for pre-survey, and the pre-survey participants were asked whether there were questions that were difficult to understand, and some of the questionnaires were modified in combination with the information and results collected in the pre-survey. The formal questionnaire distribution used a combination of online and offline survey methods, randomly distributing questionnaires and recommending applets in a hospital health check-up centre, with a total of 462 valid questionnaires recovered, of which 216 were male and 246 were female, with an age range of 25–60 years old. In terms of education level, there were 272 people in high school/secondary school and below, 131 people in bachelor’s degree/college, and 59 people in master’s degree and above. In terms of occupational distribution, there were 159 institutional government and institutions, 171 enterprise workers, and 132 freelancers and students (Table 2).

**Table 2:** Demographic characteristics of the participants.

Title	Options	Frequency count	Percentage of participants (%)
Gender	Male	216	46.75
	Female	246	53.25
Age	25–30	102	22.07
	31–40	167	36.15
	40–60	193	41.78
The educational level of the participants	High school or vocational school and below	272	58.87
	Bachelor’s degree/Associate’s degree	131	28.35
	Master’s degree and above	59	12.77
Occupation	Government agencies and public institutions	159	34.41
	Employees of enterprises	171	37.01
	Freelancers and students	132	28.57
Monthly income	Up to 2,000 yuan	82	17.75
	2,001 to 5,000 yuan	111	24.03
	5,001 to 8,000 yuan	135	29.22
	8,001 to 10,000 yuan	94	20.34
	Above 10,000 yuan	40	8.66
Your place of residence	City	391	84.63
	Suburbs	56	12.12
	Countryside	15	3.25

### 3.3 Data processing

The data processing and analyses in this paper were conducted using SPSS 26.0 and AMOS 26.0. Initially, reliability and validity were tested using the Confirmatory Factor Analysis (CFA) validated factor analysis method. In this method, reliability was measured using Cronbach’s alpha coefficient, and validity was measured using standardized factor loadings, combined reliability (CR) and average variance extracted (AVE). Secondly, the data were analyzed for model fit indicators, and the χ2 /df, CFI, TLI, RMSEA and IFI to test whether the model needed to be corrected; finally, an overall model analysis was conducted using structural equation modelling to verify whether the various research hypotheses were valid.

## 4 Results

### 4.1 Basic situation of proactive health behavior

As shown in Table 3, more than half of the participants reported only an average understanding of proactive health behavior, while nearly one-fifth expressed unfamiliarity. Regarding actual implementation, most participants indicated that they either frequently or occasionally engaged in such behaviors, suggesting a moderate level of practice in daily life. In terms of self-assessed physical and mental health, about one-third described their condition as good, another third as fair, and fewer considered it average or poor. When asked about recent illness experiences, the majority reported no illnesses, while others mentioned chronic or acute conditions, with chronic issues being more common.

**Table 3:** Basic information on participants’ proactive health behaviors.

Title	Options	Frequency count	Percentage of participants (%)
Have you previously been aware of proactive health behaviors	Very well aware	33	7.14
	Relatively aware	97	21
	Somewhat aware	251	54.33
	Not very familiar	81	17.53
Do you engage in proactive health behaviors	Frequently have	169	36.58
	Occasionally have	172	37.23
	Sometimes have	84	18.18
	Seldom have	37	8.01
How do you feel about your physical and mental health	Excellent	153	33.12
	Good	169	36.58
	Average	131	28.35
	Poor	9	1.95
Have you experienced any illnesses recently	No illness	316	68.4
	Chronic disease	124	26.84
	Acute illness	20	4.33
	Have both	2	0.43

As shown in Table 4, Pearson correlation analysis reveals that perceived susceptibility, perceived severity, perceived benefits, attitude toward proactive health behavior, subjective norm, and perceived behavioral control have significant positive correlations with proactive health behavior intention (*p* < 0.01). Among the correlations, the strongest positive association was observed between perceived severity and intention toward proactive health behavior (r = 0.531, *p* < 0.01), suggesting that individuals who recognize the seriousness of potential health risks are more likely to form intentions to engage in proactive health actions. Conversely, perceived behavioral control had a relatively weaker correlation with intention (r = 0.468, *p* < 0.01), indicating that while individuals’ confidence in their ability to take health actions is important, their perceived threat plays a stronger role in shaping behavioral intention.

**Table 4:** Analysis of the basic situation of factors driving proactive health behaviors.

Factors	Mean	Standard deviation	1	2	3	4	5	6	7	8	9
1. Perceived susceptibility	4.463	1.389	1								
2. Perceived severity	4.494	1.416	0.447**	1							
3. Perceived benefits	4.468	1.435	0.463**	0.455**	1						
4. Perceived barriers	3.376	1.492	−0.445**	−0.483**	−0.484**	1					
5. Attitude toward proactive health behavior	4.431	1.347	0.487**	0.518**	0.533**	−0.535**	1				
6. Subjective norms	4.514	1.466	0.455**	0.430**	0.446**	−0.504**	0.468**	1			
7. Perceived behavioral control	4.615	1.530	0.418**	0.448**	0.468**	−0.488**	0.435**	0.405**	1		
8. Self-efficacy	4.636	1.368	0.432**	0.463**	0.440**	−0.461**	0.451**	0.428**	0.502**	1	
9. Intention toward proactive health behavior	4.798	1.546	0.499**	0.531**	0.472**	−0.567**	0.487**	0.426**	0.468**	0.488**	1

*p < 0.05; **p < 0.01.

### 4.2 Reliability and validity test of proactive health behavior intention questionnaire

The Cronbach’s alpha coefficient was used to assess the degree of consistency between different items in the measurement instruments (Table 5). The results showed that the Cronbach’s alpha coefficient of each questionnaire was 0.825 ~ 0.901. The value of alpha coefficient ranges from 0 to 1, and the closer the value is to 1, the stronger the internal consistency is, and usually, the alpha coefficient is more than 0.7 indicating that the measurement instrument has a high degree of reliability. The results show that the internal consistency of the dimensions of the questionnaire is good. The results show that the internal consistency of the dimensions of the questionnaire is good. All constructs demonstrated strong internal consistency (Cronbach’s *α* > 0.8), indicating reliable measurement tools. Notably, the construct “perceived barriers” exhibited the highest internal reliability (α = 0.901), suggesting its robustness in reflecting psychological obstacles to proactive health behavior. The result of the KMO test is 0.937, which is greater than 0.6; the *p*-value of the Bartlett’s spherical test is less than 0.05, which indicates that the collected data of the questionnaire can be subjected to factor analysis.

**Table 5:** Reliability analysis of the proactive health intention driving factors questionnaire.

Dimension	Construct	Corrected item-total correlation (CITC)	Cronbach’s alpha if item deleted	Cronbach’s α
Attitude toward proactive health behavior	ATT1	0.765	0.78	0.852
	ATT2	0.64	0.832	
	ATT3	0.687	0.814	
	ATT4	0.687	0.813	
Subjective norms	SN1	0.752	0.71	0.831
	SN2	0.665	0.793	
	SN3	0.676	0.784	
Perceived behavioral control	PBC1	0.764	0.792	0.865
	PBC2	0.732	0.82	
	PBC3	0.736	0.817	
Intention toward proactive health behavior	AHBI1	0.818	0.816	0.891
	AHBI2	0.772	0.856	
	AHBI3	0.77	0.858	
Self-efficacy	EFF1	0.731	0.723	0.828
	EFF2	0.685	0.765	
	EFF3	0.658	0.791	
Perceived susceptibility	PS1	0.798	0.78	0.859
	PS2	0.64	0.845	
	PS3	0.678	0.83	
	PS4	0.716	0.815	
Perceived severity	PSY1	0.772	0.809	0.867
	PSY2	0.714	0.832	
	PSY3	0.694	0.84	
	PSY4	0.701	0.836	
Perceived benefits	PB1	0.746	0.698	0.825
	PB2	0.655	0.786	
	PB3	0.663	0.778	
Perceived barriers	DOP1	0.788	0.869	0.901
	DOP2	0.745	0.884	
	DOP3	0.809	0.861	
	DOP4	0.773	0.874	

Validated factor analysis was used to test the convergent validity and discriminant validity, and the AVE values of the nine dimensions were 0.595 ~ 0.733, which were all greater than 0.5, and the CR values were 0.831 ~ 0.901, which were all greater than 0.7, and they all reached the standard of eligibility. Meanwhile, the loading coefficients of each item and the corresponding factor are all greater than 0.6, indicating that the correspondence between the items and the factors is strong, and this result indicates that the convergent validity within the dimensions meets the standard. Meanwhile, by assessing the model fit of the validation factors, we observed that most of the fit indicators met the criteria, indicating that the constructed model demonstrated a good fit on both the original data and the independent validation data (Table 6). This suggests that our model accurately reflects the relationships between the variables in the data and that the use of validation data further validates the model’s ability to generalize to unseen contexts. Overall, these results suggest that the collected data reliably capture the relationships among key variables in the model and provide a robust foundation for subsequent structural modeling and interpretation.

**Table 6:** Model fit analysis of proactive health intention driving factors.

Fit indicators	Criteria	Observed value	Fit results
Absolute fit indices			
CMIN/DF	<3	1.602	Good
GFI	>0.8	0.922	Good
AGFI	>0.8	0.902	Good
RMSEA	<0.08	0.036	Good
Incremental fit indices			
NFI	>0.8	0.929	Good
IFI	>0.8	0.972	Good
TLI	>0.8	0.967	Good
CFI	>0.8	0.972	Good
Parsimonious fit indices			
PNFI	>0.5	0.795	Good
PCFI	>0.5	0.832	Good

### 4.3 Structural equation model analysis of proactive health intention drivers

After establishing the reliability and validity of the measurement instruments, a structural equation modeling (SEM) analysis was conducted to test the hypothesized relationships among key variables. Building on the validity and reliability tests, the structural equation model results (Table 7) show that the Chi-Square/df ratio (CMIN/df) is 1.752, less than 3, indicating that the model fits relatively well with the observed data. The deviation between the model and the observed data is slight. Additionally, some essential fit indices, such as the Comparative Fit Index (CFI) and Incremental Fit Index (IFI), were 0.964 and 0.964, respectively, higher than the commonly accepted threshold of 0.8. This further confirms the model is a good fit. Furthermore, other indices such as Goodness of Fit Index (GFI), Adjusted Goodness of Fit Index (AGFI), and Root Mean Square Error of Approximation (RMSEA) all showed good performance, further supporting the model’s adequacy. While all paths in the model were statistically significant, the standardized path coefficient from attitude to intention (*β* = 0.389) was the strongest, highlighting that positive attitudes toward proactive health behaviors are the most influential predictor of intention. In contrast, the weakest significant path was from subjective norms to intention (*β* = 0.183), suggesting that social influence plays a smaller but still meaningful role in shaping behavioral intention. These findings emphasize the importance of targeting individuals’ internal evaluations and self-perceptions in health interventions, alongside—but not solely relying on—social norms.

**Table 7:** Proactive health intention driving factors structural equation model statistical analysis table.

Fit indicators	Criteria	Observed value	Fit results
Absolute fit indices			
CMIN/DF	<3	1.752	Good
GFI	>0.8	0.912	Good
AGFI	>0.8	0.893	Good
RMSEA	<0.08	0.04	Good
Incremental fit indices			
NFI	>0.8	0.92	Good
IFI	>0.8	0.964	Good
TLI	>0.8	0.959	Good
CFI	>0.8	0.964	Good
Parsimonious fit indices			
PNFI	>0.5	0.808	Good
PCFI	>0.5	0.846	Good

**Figure 2:**
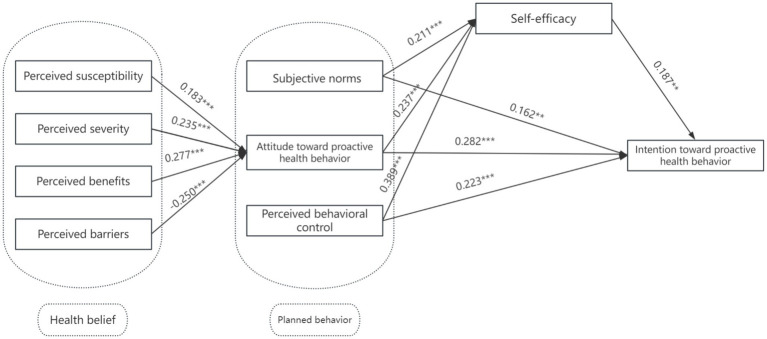
Proactive health intention driver structural equation model results.

As shown in Table 8 and Figure 2, the absolute values of the standardized path coefficients in the structural equation model range from 0.183 to 0.389, and all are statistically significant (*p* < 0.05), indicating that the relevant paths have a considerable impact.

**Table 8:** Path coefficients analysis in the structural equation model for proactive health intention factors.

Path	Standardized path coefficient	Unstandardised path coefficient	S.E.	C.R.	*p*	Hypothesis test results
Perceived susceptibility → Attitude toward proactive health behavior	0.183	0.211	0.061	3.459	***	H1a supported
Perceived severity → Attitude toward proactive health behavior	0.235	0.271	0.064	4.244	***	H1b supported
Perceived benefits → Attitude toward proactive health behavior	0.277	0.347	0.074	4.681	***	H1c supported
Perceived barriers → Attitude toward proactive health behavior	−0.25	−0.257	0.056	−4.59	***	H1d supported
Attitude toward proactive health behavior → Self-efficacy	0.237	0.177	0.044	3.972	***	H3 supported
Subjective norms → Self-efficacy	0.211	0.191	0.053	3.597	***	H3 supported
Perceived behavioral control → Self-efficacy	0.389	0.306	0.047	6.524	***	H3 supported
Attitude toward proactive health behavior → Intention toward proactive health behavior	0.282	0.301	0.062	4.872	***	H2a supported
Subjective norms → Intention toward proactive health behavior	0.162	0.209	0.073	2.889	0.004	H2b supported
Perceived behavioral control → Intention toward proactive health behavior	0.223	0.251	0.067	3.724	***	H2c supported
Self-efficacy → Intention toward proactive health behavior	0.187	0.268	0.092	2.924	0.003	H3 supported

** stands for statistical significance at *p* < 0.001.

Further insights are derived from the path analysis (Table 9), which indicates that the direct effects of proactive health behavior attitude, subjective norm, and perceived behavioral control on proactive health behavior intention are significant, ranging from 0.161 to 0.282 (p < 0.05). Among these, the effect of behavioral attitude on intention was the strongest. This suggests that individuals with a more positive evaluation of proactive health behaviors—such as believing they are beneficial, worthwhile, or important—are more likely to intend to engage in such behaviors. Therefore, promoting positive attitudes (e.g., through health education, awareness campaigns, or motivational messaging) may be an effective strategy to enhance behavioral intention. Recent research also confirms that attitude is a key determinant of intention in health-related behaviors. For instance, Karekla et al. (27) found that among various psychosocial factors, positive attitudes toward health-protective behaviors were the most consistent predictor of behavioral intention in both preventive and proactive health contexts. The indirect effects are also substantial, ranging from 0.039 to 0.073 (*p* < 0.05), and the total effects are significant, ranging from 0.201 to 0.327 (*p* < 0.05). The direct effect of self-efficacy on intention also highlights the importance of individuals’ confidence in their ability to adopt health behaviors. This implies that improving individuals’ sense of control and skills—such as through guidance, peer modeling, or goal setting—can further enhance their intention to act. This indicates that proactive health behavior attitude, subjective norm, and perceived behavioral control influence proactive health behavior intention through self-efficacy, further validating the mediating role of self-efficacy. Additionally, the path from perceived barriers to behavioral attitude was negative and statistically significant. This indicates that when individuals perceive more difficulties, such as time constraints or lack of support, they are less likely to form favorable attitudes toward proactive health behaviors. This has practical implications for intervention design—removing or reducing perceived obstacles can help foster more positive attitudes, thereby enhancing the likelihood of behavioral engagement.

**Table 9:** Mediating effect of self-efficacy.

Path	Parameter	Estimate	Lower	Upper	*p*	Hypothesis test results
Attitude toward proactive health behavior → Self-efficacy → Intention toward proactive health behavior	Direct effects	0.282	0.143	0.444	0	Supported
	Indirect effects	0.044	0.011	0.106	0.007	
	Total effects	0.327	0.183	0.493	0	
Subjective norms → Self-efficacy → Intention toward proactive health behavior	Direct effects	0.162	0.024	0.305	0.02	Supported
	Indirect effects	0.039	0.01	0.093	0.007	
	Total effects	0.201	0.059	0.345	0.005	
Perceived behavioral control → Self-efficacy → Intention toward proactive health behavior	Direct effects	0.223	0.082	0.364	0.001	Supported
	Indirect effects	0.073	0.021	0.143	0.008	
	Total effects	0.296	0.162	0.426	0	

## 5 Discussion

### 5.1 The need for improved understanding of proactive health behavior

Proactive health behavior involves actively and intentionally managing health risk factors such as labour, rest, diet, exercise, and emotional well-being. This approach emphasizes preventive action before illness occurs, rather than merely responding to symptoms or diagnoses. The findings reveal that approximately one-third of the participants reported engaging in proactive health behaviors frequently. This may be attributed to the heightened awareness of health behaviors following the end of the COVID-19 pandemic, which has led to a more proactive approach to health. Beyond individual motivations, broader societal transformations are influencing how health behaviors are understood and enacted. This trend may also reflect broader structural changes in how health is perceived socially—where personal health management becomes a form of social responsibility and even a moral imperative. Such a shift raises questions about potential inequalities in health literacy and access to health-promoting environments, which deserve further exploration. There has been a shift from passive treatment to an active health paradigm in the broader context of promoting public health and advocating for proactive health. The “2024 China Proactive Health Insight Report” suggests that 68% of respondents engage in healthy lifestyle behaviors to save on future medical or care expenses, with triggers such as societal news and advice from doctors or hospitals as motivations for adopting proactive health behaviors (28). This aligns with the increasing recognition that proactive health behavior is not only a personal responsibility but also a critical determinant of population-level health outcomes. As such, understanding the intention behind such behaviors is pivotal for shaping interventions that can effectively change behavior before illness occurs.

However, the present study also revealed that many participants reported infrequently engaging in proactive health behaviors and lacked adequate knowledge of related concepts. These findings align with data from the National Health Commission, which indicated that in 2023, only 42% of China’s urban and rural residents demonstrated basic health knowledge and concepts, and the overall health literacy level stood at 29.7% (29). Despite extensive efforts by governments and health departments to promote proactive health education and awareness, the results have not been entirely satisfactory. On the one hand, the content, formats, and communication channels for proactive health education may not be diverse or effective enough, limiting the public’s capacity to absorb and comprehend the information. On the other hand, as “proactive health behavior” is a relatively new term, the public may struggle to fully understand and accept it, resulting in suboptimal effectiveness of promotional efforts (30).

### 5.2 Health beliefs as preceding factors influencing proactive health behavior intention

The current study found that behavioral attitude had the most significant impact on proactive health behavior intention, and the four health belief factors—perceived susceptibility, perceived severity, perceived benefits, and perceived barriers—were all found to influence the attitude towards proactive health behaviors significantly. These results illustrate how individuals’ cognitive evaluations of health risks and benefits influence their attitudes and, ultimately, intentions. This is also reflected in the correlation coefficient between perceived benefits and attitude (*p* < 0.01; Table 4), suggesting that recognizing the advantages of health behaviors strongly shapes evaluative beliefs. Such findings support the notion that interventions emphasizing tangible benefits may be more persuasive than those focusing solely on risk reduction. This result is consistent with previous research integrating the health belief model and the theory of planned behavior (31, 32). Specifically, individuals who are more likely to perceive a potential health issue, view their acute or chronic health conditions as serious, believe that proactive health behaviors can prevent or mitigate health problems, and have greater confidence in their abilities and surrounding circumstances are more likely to adopt a positive attitude towards proactive health behaviors. In the theory of planned behavior, behavioral beliefs are an essential determinant of behavioral attitude and are considered external driving behavior factors (14). The four factors in the health belief model reflect an individual’s perception of health threats and the outcomes of proactive health behaviors. This finding aligns with prior theoretical and empirical research suggesting that health beliefs are antecedents of health-related attitudes. For instance, the health belief model posits that individuals’ perceptions of susceptibility, severity, benefits, and barriers shape their attitudes toward adopting health behaviors (33). Similarly, in the theory of planned behavior, behavioral beliefs—often overlapping with health beliefs—form the foundation of behavioral attitudes (34).

Results from the present study show that perceived benefits significantly impact the attitude toward proactive health behavior. This may be because proactive health behaviors are preventive, therapeutic, and active rather than passive. Therefore, when individuals recognize the benefits of engaging in such behaviors, they are more likely to be motivated to initiate proactive health actions. Perceived barriers were found to influence the attitude towards proactive health behaviors negatively. Kiely et al. (35) also found that perceived barriers were the main reason for preventing physical exercise behaviors compared to other factors in the health belief model during the pandemic. Additionally, perceived severity and perceived susceptibility also had significant effects. Trifiletti et al. (36) argued that both perceived susceptibility and severity indicate perceived threat and can influence the occurrence of health behaviors, especially when the sample is sick or at risk of epidemics, and the effect on related behaviors is more pronounced.

### 5.3 Attitude as an important factor influencing proactive health behavior intention

Given the foundational role of attitudes in shaping behavioral intention, further analysis is needed to understand how they operate within the proactive health context. Although the public has a certain level of understanding and experience with proactive health behaviors, there is still considerable room for improvement. It is essential to promote proactive health behaviors to shift from a disease-centred approach to a health-centred approach. Findings indicate that participants’ attitudes, subjective norms, and perceived behavioral control significantly and positively influenced proactive health behavior intention. This result is consistent with previous studies that have applied the theory of planned behavior to explore behavioral intentions in domestic and international contexts (37, 38). This indicates that improving the public’s positive perception and attitude towards proactive health behaviors, providing convenient equipment, venues, and guidance for participation, and creating a relaxed and accessible environment where the public can recognize the significance of engaging in proactive health behaviors can help enhance their confidence and ultimately promote the formation of proactive health behavior intentions.

Among all the pathways influencing proactive health behavior intention, the standardized regression coefficient of proactive health behavior attitude on intention is the highest, highlighting the critical role of behavior attitude. This is consistent with previous research on the factors influencing health behavior intention (39). This may be because individuals who believe that engaging in proactive health behaviors is beneficial for enhancing physical health, improving emotional states, and increasing the quality of life are more likely to intend to engage in proactive health behaviors. These findings reinforce the pivotal influence of individual beliefs in shaping health-related decisions. In practical terms, this suggests that public health campaigns should highlight the tangible personal benefits of proactive health actions—such as better sleep, reduced fatigue, or emotional resilience—to reinforce positive attitudes and motivate intention formation. Behavioral attitude is an individual’s overall evaluation of behavior based on their perception of the outcomes and benefits of that behavior. A positive behavioral attitude strengthens the intention to engage in the behavior (40).

The study also found that perceived behavioral control is another important influencing factor of proactive health behavior intention. Previous studies on health-related behavioral intentions have supported this view. For instance, Addis et al. (41) found that perceived behavioral control was a key determinant of exercise intention among pregnant women. Similarly, Dhaliwal and Campbell (42) reported that both attitude and perceived control significantly influenced physical activity intention during pregnancy. These findings highlight the robustness of the theory of planned behavior across health contexts (41, 42). This suggests that if the public feels confident about engaging in proactive health behaviors, they are more likely to have higher proactive health behavior intentions. While perceived behavioral control enhances confidence in action, however, confidence alone may not suffice. Practical programs should integrate motivational elements with structural supports—such as app-based prompts, incentive schemes, or community health campaigns—to bridge the gap between intention and action (43). In real-world application, the significant effect of self-efficacy on intention underscores the need to design health programs that build individuals’ confidence—through achievable goal-setting, peer modeling, and positive feedback mechanisms. When people feel capable of executing health behaviors, they are more likely to form a strong behavioral intention (18). Therefore, promoting proactive health behavior intention should consider the convenience of venues, tasks, and equipment. When the public can overcome obstacles such as time, location, and task difficulty, their intention to engage in proactive health behaviors may increase. This finding is also consistent with the assumptions of the theory of planned behavior, whereby the public tends to have a high degree of control over the behaviors they initiate or push themselves to engage in (19).

The influence of subjective norms on proactive health behavior intention reached significance, but the path coefficient value was the lowest. This suggests that the respondents’ friends, family, or colleagues influence their proactive health behavior intention, but the impact is limited. Many studies have found similar results on physical exercise and other health behaviors. For example, research by Gomes et al. (40) and Godin et al. (44) indicated that the effect of subjective norms is weak and, in some cases, insignificant. If those around individuals do not fully recognize the value of a particular behavior or if there is no significant environmental pressure to engage in that behavior, the influence of subjective norms may be diminished (45). The public’s understanding of proactive health is still limited, so the surrounding environment may not yet exert enough pressure to encourage proactive health behaviors.

Moreover, the significant negative impact of perceived barriers on behavioral attitude observed in our model suggests an important practical implication: when individuals perceive obstacles—such as lack of time, insufficient knowledge, limited access to resources, or unsupportive social environments—their attitudes toward proactive health behavior tend to worsen. This underlines the need for interventions not only to enhance motivation but also to reduce real or perceived barriers. Examples include offering time-efficient health plans, improving public access to exercise facilities, simplifying health education content, and building supportive community networks to foster more favorable attitudes.

### 5.4 Self-efficacy as a mediator of proactive health behavior intention

In the present study, attitude, subjective norms, and perceived behavioral control directly influence proactive health behavior intention and indirectly affect behavioral intention through the mediating role of self-efficacy. The path coefficients for the influence of attitude, subjective norms, and perceived behavioral control on self-efficacy were significant. Self-efficacy, which refers to one’s confidence in performing proactive health behaviors, increases when individuals have more positive attitudes toward proactive health behaviors, receive more excellent support from significant others, and find the environment more convenient (46). The path coefficient for the influence of self-efficacy on proactive health behavior intention was also substantial, indicating that the stronger individuals’ belief in their ability to achieve the expected health outcomes through proactive health behaviors, the more likely they are to form proactive health behavior intentions. This view is widely supported in recent research related to health behavior promotion. For example, Xu et al. (6) demonstrated that self-efficacy significantly predicted retirees’ participation in health screenings when analyzed through an extended theory of planned behavior framework. Similarly, Lu et al. (47) found through a meta-analysis that eHealth interventions targeting children were effective in enhancing self-efficacy, which in turn led to improved physical activity behaviors (47, 48). High self-efficacy facilitates emotional regulation, increases behavioral motivation and confidence, and helps individuals overcome barriers to action (49). Self-efficacy has been strongly associated with habitual physical exercise (50). Like exercise habits, proactive health behaviors are often deliberate and goal-oriented. Therefore, if individuals believe they can achieve beneficial results from proactive health behaviors, they are more likely to develop proactive health behavior intentions.

### 5.5 Limitations and prospects

While the findings offer meaningful insights, it is important to acknowledge several limitations. It is imperative to acknowledge the limitations of this study, which are as follows: First, the sampling method and sample size are limited. The sample was collected from only one health checkup centre, which may affect the generalizability of the findings. Although the study recruited participants from a single health checkup center, the sample may not fully represent the diversity of broader populations across different regions or healthcare settings. This single-site data collection limits the generalizability of the findings, as contextual factors—such as local health infrastructure, cultural norms, and public health outreach—may influence individuals’ health behavior intentions differently. Second, the structural equation model did not include demographic variables, such as health conditions, which could yield different results if incorporated. In addition, although the sample was relatively large, it was also highly heterogeneous in terms of age, occupation, education, and health status. Such diversity may have introduced uncontrolled variance into the results, potentially obscuring subgroup-specific patterns or weakening the precision of some estimates. Future studies should consider stratified or subgroup analyses, or recruit more homogeneous populations when targeting specific intervention designs. Third, the cross-sectional nature of the questionnaire used in this study limits the possibility of establishing causal relationships between variables. Moreover, although the structural equation model demonstrated a good overall fit, its static nature restricts our ability to observe temporal changes or contextual shifts in intention formation. Future studies could incorporate longitudinal designs or ecological momentary assessment (EMA) to capture dynamic processes in real-time. In addition, the study did not incorporate specific control measures for potential response biases, such as social desirability bias or acquiescence bias, which may have influenced participants’ self-reported behaviors and attitudes. Future research could consider applying techniques such as validity scales, indirect questioning, or anonymity reinforcement to mitigate these biases. To address these issues and enhance generalizability, future research should consider expanding the sample size, conducting a separate study on a specific population group, or utilizing a longitudinal questionnaire to examine the presence of cross-sectional effects.

## 6 Conclusion

This study investigated the key factors influencing proactive health behavior intention by integrating the health belief model and the theory of planned behavior into a unified model. The findings revealed three main conclusions: First, perceived susceptibility, severity, benefits, and barriers were all significant antecedents of proactive health behavior, with perceived benefits having the strongest effect.

Second, attitude, subjective norms, and perceived behavioral control directly and positively predicted behavioral intention, with attitude being the most influential factor. Third, self-efficacy partially mediated the effects of these three variables on intention, highlighting its important but not exclusive role in the mechanism.

Based on these findings, the following recommendations are proposed: firstly, to strengthen the promotion of active health-related content, especially to enable the public to fully understand the possible benefits of proactive health behaviors, and to increase the environment conducive to proactive health behaviors. Secondly, focus on cultivating the public’s attitude towards active health behaviors and establishing the public’s concept of proactive health as soon as possible. Finally, we should focus on improving the public’s self-efficacy in proactive health behaviors, which can be achieved through goal-setting, social support, professional guidance and other methods to enhance the public’s confidence in proactive health behaviors.

## Data Availability

The datasets presented in this study can be found in online repositories. The names of the repository/repositories and accession number(s) can be found in the article/supplementary material.
